# Residual Self-Calibration and Self-Attention Aggregation Network for Crop Disease Recognition

**DOI:** 10.3390/ijerph18168404

**Published:** 2021-08-09

**Authors:** Qiang Zhang, Banyong Sun, Yaxiong Cheng, Xijie Li

**Affiliations:** 1 School of Science, Wuhan University of Technology, Wuhan 430070, China; zhangqiang977966@yeah.net (Q.Z.); drchern@yeah.net (Y.C.); 2 Key Laboratory of Spectral Imaging Technology, Xi’an Institute of Optics and Precision Mechanics of CAS, Xinxi Road No. 17, Xi’an 710119, China; hhhhaas@yeah.net

**Keywords:** crop disease recognition, self-calibration, self-attention, residual

## Abstract

The correct diagnosis and recognition of crop diseases play an important role in ensuring crop yields and preventing food safety. The existing methods for crop disease recognition mainly focus on accuracy while ignoring the algorithm’s robustness. In practice, the acquired images are often accompanied by various noises. These noises lead to a huge challenge for improving the robustness and accuracy of the recognition algorithm. In order to solve this problem, this paper proposes a residual self-calibration and self-attention aggregation network (RCAA-Net) for crop disease recognition in actual scenarios. The proposed RCAA-Net is composed of three main modules: (1) multi-scale residual module, (2) feedback self-calibration module, and (3) self-attention aggregation module. Specifically, the multi-scale residual module is designed to learn multi-scale features and provide both global and local information for the appearance of the disease to improve the performance of the model. The feedback self-calibration is proposed to improve the robustness of the model by suppressing the background noise in the original deep features. The self-attention aggregation module is introduced to further improve the robustness and accuracy of the model by capturing multi-scale information in different semantic spaces. The experimental results on the challenging 2018ai_challenger crop disease recognition dataset show that the proposed RCAA-Net achieves state-of-the-art performance on robustness and accuracy for crop disease recognition in actual scenarios.

## 1. Introduction

The occurrence of crop diseases has a certain negative impact on agricultural production. If crop diseases are not discovered in time, it will increase the risk of food loss [1], especially for some major food crops, such as corn, rice, wheat, etc., which are key to meeting human living needs and promoting productivity development. Therefore, it is of great practical significance to explore an intelligent, low-cost, and highly accurate method to implement crop disease recognition. The feature extraction and pattern recognition in machine learning help to identify the type and severity of crop diseases. Automatic quality analysis of plant health status through the color, shape and size of plant leaf images is an accurate and reliable method to improve productivity [2,3].

Crop disease recognition based on traditional image processing methods is incomparable in recognition accuracy and robustness compared to methods based on deep neural networks that have emerged in recent years. Most of the current methods based on deep neural networks are trained on the public dataset PlantVillage [4] or simple background images to construct models for realizing crop disease image recognition. However, the type of method creates some problems. The public dataset PlantVillage has a simple background, and the characteristics of crop diseases are diverse. Since the acquisition of annotated images requires the participation of experts, the categories are often unbalanced, and the direct migration of the model trained on PlantVillage is not very good. When the disease recognition method based on simple background images is applied to recognize the crop disease in the actual environment, it needs to fight against various noise interference factors. In addition, the actual recognition accuracy will be greatly reduced, which cannot meet the practical requirement.

Aiming at the characteristics of crop disease image recognition with a complex background, more interference, and diverse disease features, this paper takes both the recognition accuracy and robustness of the model into account and proposes a residual self-calibration and self-attention aggregation network (RCAA-Net) for crop disease recognition in actual scenarios. The main contributions of this paper are as follows:

- A residual self-calibration and self-attention aggregation network is proposed for crop disease recognition in actual scenarios. For the problem of crop disease recognition in actual scenarios, the proposed RCAA-Net can achieve a double improvement of accuracy and robustness.
- A feedback self-calibration module is proposed to further suppress the background noise in the original deep features by fine filtering and adjusting the network features again, thereby effectively improving the robustness of the model.
- A self-attention aggregation module is proposed to automatically focus on discriminative regions by capturing multi-scale information in different semantic spaces, thereby further improving the robustness and accuracy of the model.

The rest of this paper is arranged as follows. Section 2 summarizes the related work; Section 3 details the proposed RCAA-Net method; Section 4 introduces the experimental settings and results; Section 5 gives the conclusions.

## 2. Related Work

Crop disease image recognition is a comprehensive use of image processing, phytopathology, pattern recognition and other technical means to analyze disease image information to obtain the characteristic representation and classification model of the disease so as to accurately classifying the disease category. According to the current idea of disease image recognition, methods can be divided into the following two categories.

### 2.1. Traditional Image Processing Methods

Many previous works have considered the problem of image recognition and apply a special classifier to discern healthy and diseased images. Generally speaking, plant leaves are primary information for the recognition of crop diseases because most of the symptoms of diseases first appear on leaves. In the past few decades, the recognition and classification of major diseases have been widely used in plants, including K-Nearest Neighbor (KNN) [5], Support Vector Machine (SVM) [6], Fisher Linear Discriminant (FLD) [7], Artificial Neural Network (ANN) [8], Random Forest (RF) [9], etc. The disease recognition rate of classical methods largely depends on the lesion segmentation of various algorithms and hand-crafted features, such as seven invariant moments, scale-invariant feature transform (SIFT), Gabor transform, global-local singular values and sparse representation [10,11,12]. However, hand-crafted features require expensive resource conditions and professional knowledge, and at the same time, have a certain degree of subjectivity. Moreover, it is difficult to determine which disease recognition features are the best and most robust from the extracted target. In addition, most methods cannot effectively separate leaves and lesion images from the background under complex conditions, resulting in failure to predict the occurrence of disease. Therefore, due to the complexity of diseased leaf images, automatic recognition of crop disease is still a challenging task.

### 2.2. Deep Neural Network Methods

In recent years, deep learning techniques, especially convolutional neural networks (CNN) [13,14,15], are rapidly becoming the preferred method to overcome the above-mentioned challenges [16,17,18,19,20]. Due to the scale invariance of the convolutional neural network, the image problem it solves is not limited by the scale and shows outstanding ability in recognition and classification. For example, Mohanty et al. [21] trained a deep learning model to identify 14 crops and 26 crop diseases. Ma et al. [22] used deep CNN to identify the symptoms of cucumber downy mildew, anthracnose, powdery mildew and target leaf spot, with a recognition accuracy of 93.4%. Kawasaki et al. [23] proposed a CNN-based cucumber leaf disease recognition method, which achieved an accuracy of 94.9%. Similarly, this paper also uses CNN to extract plant leaf disease characteristics and proposes a lightweight convolutional network based on VGG-16. First of all, the original network introduces depthwise separable convolution (DSC) [24] and global average pooling (GAP) [25] to replace the standard convolution operation and perform the complete operation at the end of the network. The connection layer is replaced, and at the same time, batch normalization technology is applied to training the network and improving the data distribution in the middle layer and increasing the convergence speed [26]. The experimental results of the improved network on the plant leaf disease dataset PlantVillage show that the proposed lightweight convolutional network has a significant improvement in recognition accuracy and efficiency and is suitable for the task of plant leaf disease recognition, which has strong engineering practicality and high research value. Most of these methods are aimed at PlantVillage or simple background image recognition. When facing the recognition of complex background and various noise interference in the actual environment, the recognition accuracy will often be greatly reduced due to the complex background noise interference. Therefore, improving the accuracy, robustness and anti-interference ability of crop disease image recognition in the actual environment has become the key to crop disease recognition.

## 3. Methods

This paper aims to build a novel deep convolutional neural network with simple, accurate, robust and strong anti-interference ability to achieve high-precision recognition of crop disease in images. This section first introduces the framework of the proposed RCAA-Net. Then, the multi-scale residual module, feedback self-calibration module and self-attention aggregation module are elaborated, respectively. Finally, the network parameters of the proposed RCAA-Net method are reported, and the loss function is provided.

### 3.1. Overview

**Figure 1:**
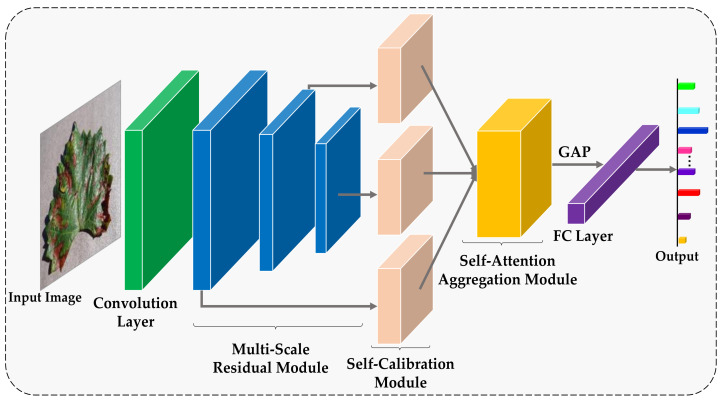
The framework of the proposed RCAA-Net.

The proposed RCAA-Net method realizes the accurate recognition of crop diseases and, meanwhile, takes the anti-interference ability into account. The overall network structure of the RCAA-Net method is shown in Figure 1. For the disease image to be classified, this paper adopts 1 convolutional layer, 3 residual modules, 3 parallel feedback self-calibration modules, 1 self-attention aggregation module, 1 global average pooling layer and 1 Softmax layer to directly output the category probability of the cropped image.

In Figure 1, in order to effectively utilize features of different scales, the output of the three residual modules is adopted in the proposed RCAA-Net method. By synthesizing the features of the three scales, it can provide richer features for the subsequent network layer, improve the recognition accuracy of the model, and indirectly improve the robustness of the network. In order to finely filter the image features to improve their anti-interference ability, for each scale feature, we input a feedback self-calibration module to finely filter the image features and improve anti-interference abilities. In addition, the three scale features processed by the feedback self-calibration module are input to the self-attention aggregation module to capture multi-scale information in different semantic spaces to automatically focus on the discriminative regions, thereby further improving the robustness and accuracy of the model. The three main modules in the proposed RCAA-Net are described in detail as follows.

### 3.2. Multi-Scale Residual Module

Residual network [27] has achieved satisfying results on IRSVRC. The residual network can not only speed up the network fitting and improve the recognition accuracy, but also has a certain anti-interference ability. Prior to this, residual networks for crop disease recognition had not attracted enough attention and research. In addition, multi-scale features can use different levels of semantic information at the same time, thereby avoiding the adverse mesoscale effects in crop disease recognition. To this end, we designed a multi-scale residual module to effectively solve the above problems and improve the performance of the crop disease recognition model.

**Figure 2:**
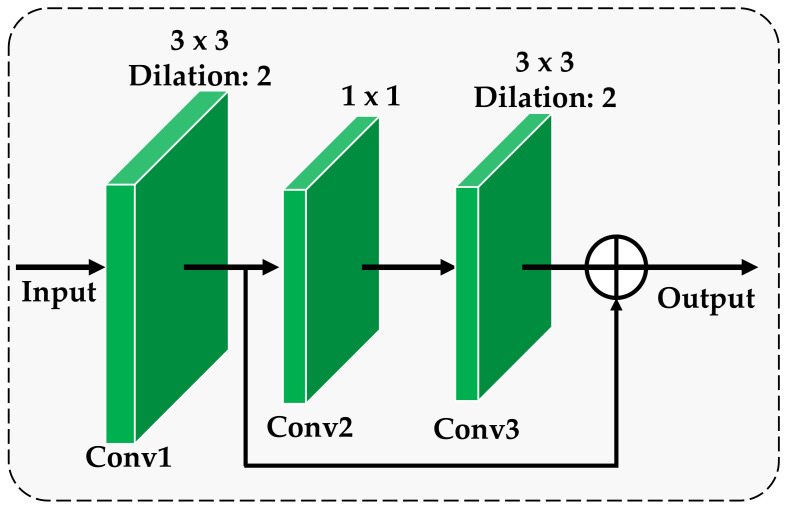
The architecture of the residual block.

The proposed multi-scale residual module consists of three consecutive residual modules, and the structure of each residual block is shown in Figure 2. As can be seen from Figure 2, each residual block consists of 3 dilation convolutional layers, which are respectively denoted as Conv1, Conv2 and Conv3. The detailed parameters of each convolutional layer are listed in Figure 2. Here, the adoption of dilation convolution is to increase the receptive field of feature points, thereby handling large-scale variance of the lesion area for crop disease without introducing additional computation [28]. The output after the input passes through Conv1, Conv2 and Conv3 are denoted as $X_{1}$, $X_{2}$ and $X_{3}$, respectively. We directly cascade $X_{1}$ and $X_{3}$ as the total output of the entire residual block. The specific cascade model can be expressed by:

(1) $$R(X_{1})=X_{1}+X_{3}=\Gamma (X_{1})+X_{1},$$

where Γ(⋅) represents the residual mapping function and R(⋅) represents the output of each residual block.

The residual block obtains more prominent fine information in the image by learning the residual mapping function. The residual block realizes that the low-level features extracted through Conv1 convolution and the high-level detailed features acquired through Conv1, Conv2 and Conv3 three-layer convolution are transmitted to the following network at the same time, and more refined feature extraction is continued. By inputting the output of the three residual blocks as multi-scale features into the subsequent network, the detailed description of the low-level features and the abstract representation of the high-level features in the convolutional neural network can be comprehensively used to provide rich and detailed feature representation for the appearance of the disease. In this way, the recognition accuracy of the model can be effectively improved.

### 3.3. Feedback Self-Calibration Module

**Figure 3:**
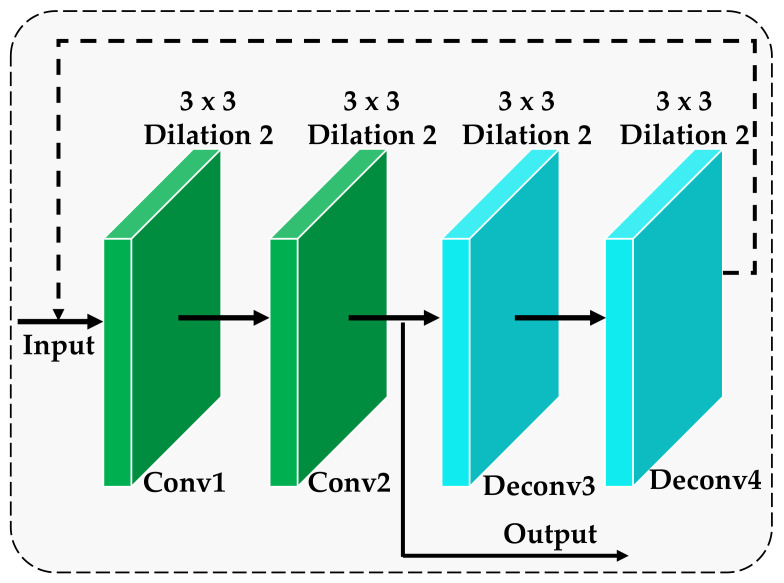
The architecture of the feedback self-calibration module.

In order to achieve high recognition accuracy and anti-interference ability for the images collected in the actual environment that may contain various noise factors, a feedback self-calibration module is designed, and its structure is shown in Figure 3. The feedback self-calibration module is to reload the convolutional layer, perform two deconvolution operations after loading, and then return the deconvolution result to the previous shallow layer. Subsequently, it is passed as output to the subsequent network layer model after repeated loading. The convolutional layer involved is a 3×3 convolution kernel with a step size of 1. We can clearly see the entire process of feedback to the self-calibration module from Figure 3. Let the input of the feedback self-calibration module be $X_{c}$, and the result after deconvolution be $X_{dec}$. Then, the feedback self-calibration module can be optimized under the constraints of the following equation:

(2) $$l_{c}={\left\|\Psi (X_{c})-X_{c}\right\|}_{2}={\left\|X_{dec}-X_{c}\right\|}_{2},$$

where Ψ(•) represents the feedback self-calibration function and ${\left\|•\right\|}_{2}$ represents the L2 norm. Through this constraint, the features after deconvolution can be used to feedback and adjust the original deep features, thereby suppressing the background noise in the original deep features and improving the robustness of the model.

In summary, the purpose of introducing the feedback self-calibration module in this paper is to return the features of the deep convolutional layer in the network to the shallow convolutional layer so that the network features can be fine-filtered and readjusted. In this way, the background noise in the original deep features is further suppressed, effectively improving the robustness of the model.

### 3.4. Self-Attention Aggregation Module

**Figure 4:**
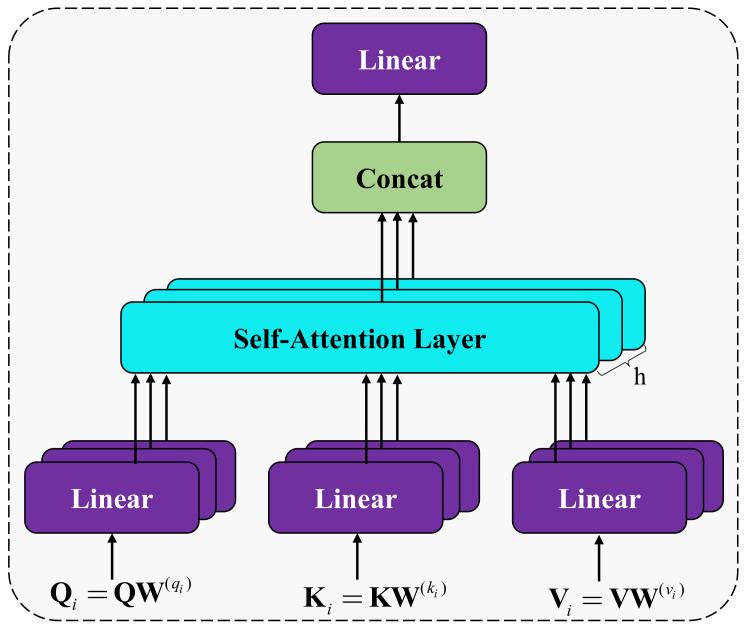
The architecture of the self-attention aggregation module.

Research has found that attention can selectively focus on important information in the data. This paper takes this as inspiration and draws on the Transformer model [29]. The multi-head self-attention mechanism (MHA) is adopted to extract dependency relationships in different semantic spaces. The architecture is shown in Figure 4. Multi-head self-attention is based on the principle of scaled dot-product attention, and its calculation formula is as follows:

(3) $$\hbar (Q,V,K)=\mathrm{softmax}\left(\frac{QK^{T}}{\sqrt{d_{k}}}\right)V$$

where ℏ(⋅,⋅,⋅) stands for scaled dot-product attention operation, Q,V,K are the query, value, and key matrix for calculating self-attention, respectively. $QK^{T}$ is the attention matrix, weighting the V matrix. $d_{k}$ represents the dimension of the key. $\sqrt{d_{k}}$ turns the attention matrix into a standard normal distribution so that the result is more stable and a balanced gradient can be obtained when backpropagating.

Based on the scaled dot-product attention calculated by Equation (1), the semantic features are integrated from the subspace containing different semantic information.

Furthermore, the value of MultiHead is obtained through the following two steps.

(1) Firstly, the Q,V and K matrices are mapped into multiple subspaces:
  (4) $$\left\{\begin{array}{c} Q_{i}=QW^{\left(q_{i}\right)}\\ K_{i}=KW^{\left(k_{i}\right)}\\ V_{i}=VW^{\left(v_{i}\right)} \end{array}\right.,$$
  where $Q_{i}$, $K_{i}$, and $V_{i}$ are the query, key and value matrix of each subspace. $W^{\left(q_{i}\right)}$, $W^{\left(k_{i}\right)}$ and $W^{\left(v_{i}\right)}$ are conversion matrices.
(2) Secondly, the scaled dot-product attention in each subspace is calculated in parallel, and then the results are concatenated to obtain the context matrix after linear transformation:
  (5) $${\mathrm{head}}_{i}=\hbar (Q_{i},K_{i},V_{i})$$
  (6) $${\mathrm{MultiHead}=\mathrm{Concat}(\mathrm{head}}_{1}{,\mathrm{head}}_{2},\ldots {,\mathrm{head}}_{h})W^{T}$$
  where ${\mathrm{head}}_{i}$ is the scaled dot-product attention of each subspace, and MultiHead is the final result.

### 3.5. Network Parameters and Loss Function

In this paper, RCAA-Net is a simple, accurate and highly robust convolutional neural network. Table 1 lists the main parameters in this method. Among them, the parameters of the multi-scale residual module and the feedback self-calibration module are shown in Figure 2 and Figure 3, respectively. In order to reduce the number of network parameters, we only use two types of kernels, 1×1 and 3×3, which also helps to avoid overfitting due to the small image set.

In order to realize the proposed RCAA-Net for end-to-end training, the objective function of this paper adopts Softmax, and its formula is as shown in Equation (7).

(7) $$l_{cls}=-\frac{1}{L}\left[\sum_{l=1}^{L}\sum_{k=1}^{K}q(y_{l}==k)\log\frac{e^{\theta_{k}^{T}X_{t}}}{\sum_{p=1}^{K}e^{\theta_{p}^{T}X_{t}}}\right]$$

where $\left(X_{1},y_{1}),(X_{2},y_{2}),\ldots ,(X_{L},y_{L}\right)$ is the training set, $X_{l}$ is the l-th training sample, and $y_{l}\in 1,2,3,\ldots ,K$ is the label corresponding to $X_{l}$. $\theta_{k}^{T}$ and $\theta_{p}^{T}$ denote the transposition of $\theta_{k}$ and $\theta_{p}$, respectively. L and K denote the number of training samples and the number of categories, respectively. q(⋅) is the guiding function.

By combining Equations (2) and (7), the final loss function is obtained as follows:

(8) $$l_{final}=l_{c}+l_{cls}={\left\|X_{dec}-X_{c}\right\|}_{2}-\frac{1}{L}\left[\sum_{l=1}^{L}\sum_{k=1}^{K}q(y_{l}==k)\log\frac{e^{\theta_{k}^{T}X_{t}}}{\sum_{p=1}^{K}e^{\theta_{p}^{T}X_{t}}}\right]$$

By minimizing the final loss in Equation (8), the proposed RCAA-Net is trained end-to-end.

## 4. Experiment

### 4.1. Experiment Setup

#### 4.1.1. Dataset

**Figure 5:**
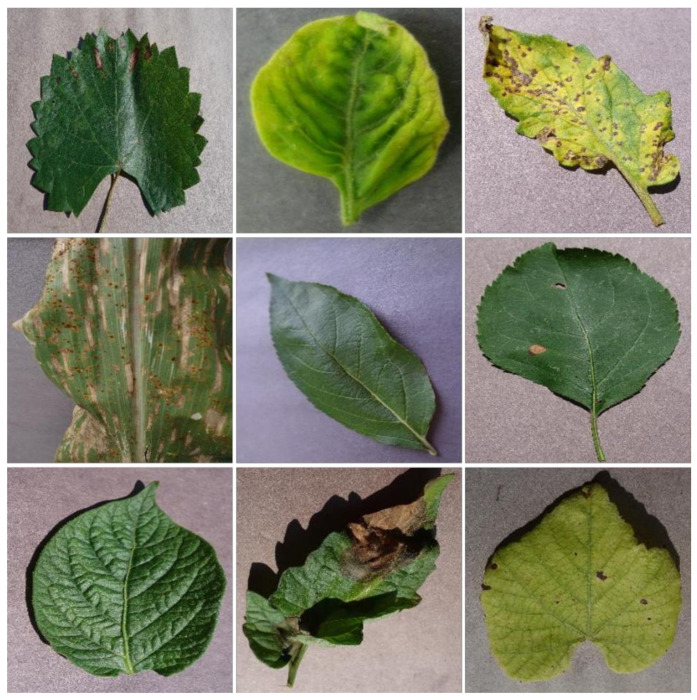
Some examples of the dataset.

The dataset employed in this paper comes from the crop disease detection competition in 2018ai_challenger. The dataset contains 31,718 training images, 4540 verification images, and 4514 testing images, covering different diseases in apples, corn, grapes, citrus, peaches, peppers, potatoes, strawberries, tomatoes and others. Some examples of the dataset are shown in Figure 5. The images in this dataset contain various noises and environmental factors such as angles and lighting. Therefore, the dataset can truly reflect the current status of data resources during crop disease image recognition in the actual environment and is sufficient to verify the accuracy and robustness of the method in this paper for crop disease recognition in actual scenarios.

#### 4.1.2. Implementation Details

We verify the RCAA-Net method on the 2018ai_challenger crop disease recognition dataset. The model is trained on a machine with NVIDIA GPU 1080i with 300 epochs. Generally, after 50 iterations of training, RCAA-Net can output satisfactory accuracy. In this paper, the Adam optimization algorithm is used to optimize the loss function of Equation (8), and the initial learning rate is $3\times 10^{-3}$. The batch size is set to 128. In the test phase, in order to prove the anti-interference ability of the proposed RCAA-Net network, we add different levels of Gaussian and salt and pepper noise to the test images to verify the recognition accuracy of the network and evaluate the robustness of the network.

#### 4.1.3. Comparison Methods

In order to verify the effectiveness and superiority of the proposed RCAA-Net, we conducted experiments on the 2018ai_challenger crop disease recognition dataset. Specifically, detailed experiments were conducted to verify the proposed RCAA-Net in terms of accuracy and robustness.

The comparison methods used in the experiment include LeafSnap SVM (RBF) method [30], LeafSnap NN method, HCF SVM (RBF) method [31], HCF-Scale Robust SVM (RBF) method [31], combined linear SVM method [31] and SIFT linear SVM method [32].

Among them, the LeafSnap NN method uses neural networks to classify and recognize gist features. HCF SVM (RBF) classifier leverages SVM (RBF) to classify hand-designed features. Here, the SVM (RBF) method is to apply the radial basis kernel function SVM classifier to classify the leaf gist features [31]. The SVM classifier was implemented by libsvm [33]. The HCF-Scale Robust SVM (RBF) method extracts the features except for the leaf contour length, area and skeleton length from the HCF features and uses the SVM (RBF) classifier for classifying. The features in the combined linear SVM method include the features extracted by the convolutional neural network ConvNet [34] and the features extracted by the HCF-Scale Robust method. Among them, ConvNet includes 5 convolutional layers, 3 maximum pooling layers and 2 fully connected layers. The SIFT linear SVM method is to extract SIFT features and use a simple linear SVM classification method based on sparse coding linear space pyramid matching SPM kernel for classification and recognition.

In addition, to make a fair comparison, we adopt the proposed RCAA-Net by adopting the input image size 256×256, which matches the input images size of the other comparison methods. The adapted method is noted as RCAA-Net (adaptive).

In order to verify the effectiveness of each module, we design different models. Specifically, a model that does not include residual connections, feedback self-calibration and self-attention aggregation is used as a baseline. On this basis, we have added residual connections to form a comparison method named RES. The feedback self-calibration module is added based on the baseline to form the self-calibration method. The self-attention aggregation module is added on the basis of the baseline to form the self-attention method. The effectiveness of each module is illustrated by comparing each method.

### 4.2. Comparison with State-of-the-Art Methods

**Figure 6:**
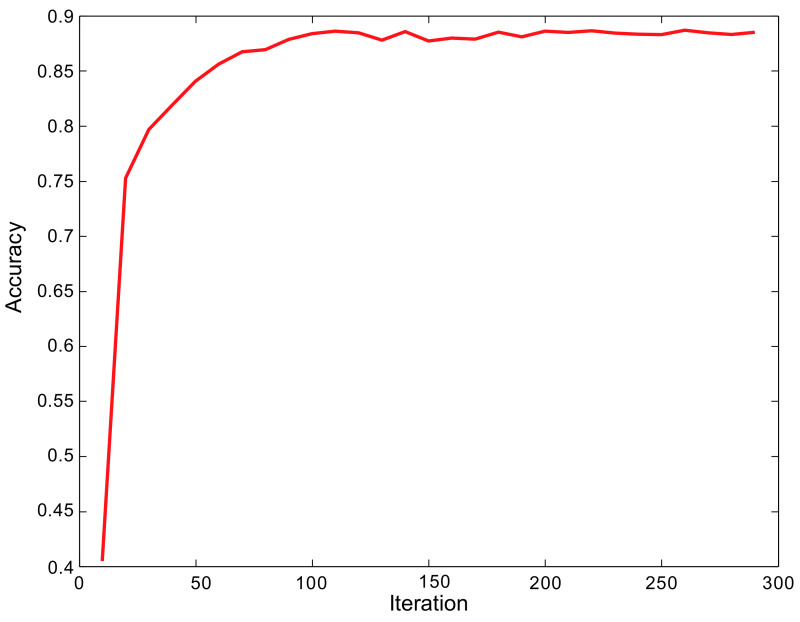
The accuracy curve during the training phase.

In order to verify the accuracy of the RCAA-Net method in this paper, this paper trains the model on the 2018ai_challenger crop disease recognition dataset. The accuracy change of the model during training is shown in Figure 6. It can be seen that the RCAA-Net proposed in the end-to-end training manner can quickly converge and achieve an ideal recognition accuracy. This shows the efficiency of the proposed RCAA-Net.

In order to more accurately illustrate the superiority of the proposed RCAA-Net method, we have compared the recognition accuracy with the existing methods. The experimental comparison results are shown in Table 2.

It can be seen from Table 2 that the RCAA-Net method in this paper is significantly better than other methods except for the combined linear SVM method. Compared with the combined linear SVM method that utilizes more complex networks, the proposed RCAA-Net method still has higher recognition accuracy, which further illustrates the efficiency of the proposed RCAA-Net method in crop disease recognition. The proposed RCAA-Net method only adopts a simple network to achieve end-to-end recognition. The size of the convolutional layer and the number of parameters are small, which effectively reduces the difficulty of model training. In this paper, the network operation parameters are only 45.68% of the ConvNet parameters in the combined linear SVM method. In addition, when identifying crop diseases in the actual environment, due to the limited number of labeled image sets and fewer parameters, the over-fitting problem caused by insufficient training data can be better alleviated by the proposed method. We argue that the main reason for the state-of-the-art performance includes two aspects. On the one hand, we develop a self-attention aggregation module to automatically focus on the discriminative regions by capturing multi-scale information in different semantic spaces, which can effectively make the proposed RCAA-Net more accurate. On the other hand, we develop a feedback self-calibration module for further suppressing the background noise in the original deep features by fine filtering and adjusting the network features, thereby effectively improving the effectiveness of the proposed RCAA-Net. Note that when we adopt more small-input images (224×224), the accuracy of the proposed method had almost no change, but the computational burden was further decreased.

### 4.3. The Discussion under Different Noise Conditions

In order to prove the effectiveness of various modules in the proposed RCAA-Net method, we perform the proposed RCAA-Net method, baseline model, RES model, self-calibration model and self-attention model on the 2018ai_challenger crop disease recognition dataset. The experimental results are shown in Table 3.

From Table 3, it can be seen that on the 2018ai_challenger crop disease recognition dataset, when the general convolutional network CNN is utilized, the recognition accuracy rate was 0.617. When the residual connection, the feedback self-calibration module and self-attention aggregation module were added separately, the accuracy rates were increased to 0.705, 0.684 and 0.751, respectively. Compared with the baseline, RES, self-calibration and self-attention methods, the RCAA-Net method in this paper achieves the highest recognition accuracy rate of 0.892. This fully shows that when combined with the residual connection, the self-calibration module and the self-attention aggregation module can be fed back to bring higher recognition accuracy, which has important enlightening significance and reference value for crop disease recognition and recognition problems in other fields.

Furthermore, in order to verify the robustness of the proposed RCAA-Net method in this paper, we add Gaussian noise, salt and pepper noise, and Gaussian and salt and pepper noise at the same time to the testing set. The proposed RCAA-Net method is evaluated to show the robustness of the model by adding different levels of noise interference. The noise level interval added in the testing set of this experiment is 0.005.

**Figure 7:**
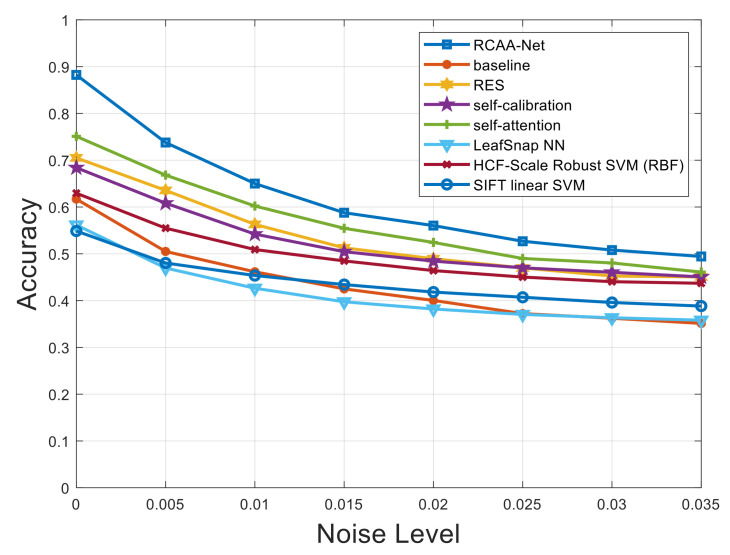
Recognition accuracy results when adding different levels of Gaussian noise.

**Figure 8:**
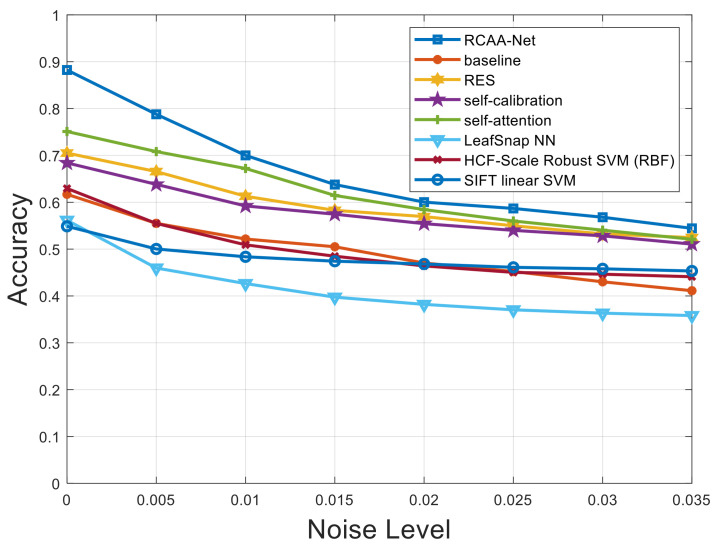
Recognition accuracy results when adding different levels of salt and pepper noise.

**Figure 9:**
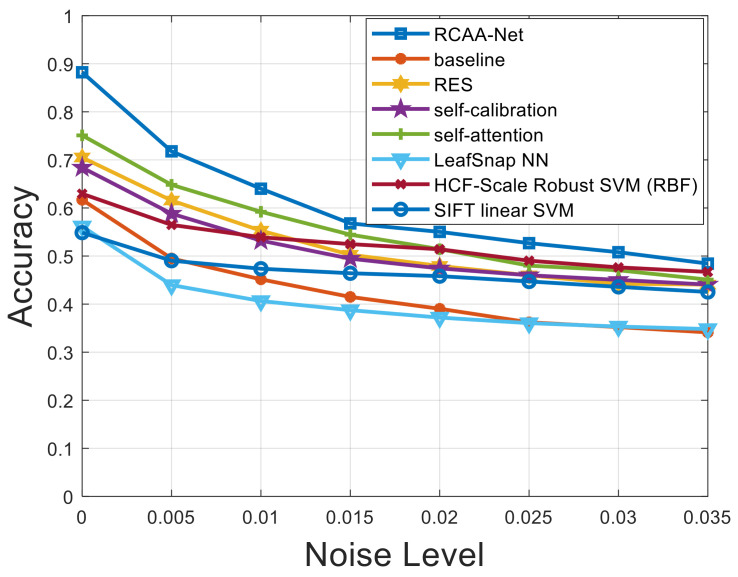
Recognition accuracy results when adding different levels of Gaussian noise + salt and pepper noise.

Figure 7, Figure 8 and Figure 9 respectively show the comparison results of recognition accuracy when adding Gaussian noise, salt and pepper noise, and both Gaussian and salt and pepper noise in the test set of the 2018ai_challenger crop disease recognition dataset. In Figure 7, Figure 8 and Figure 9, the abscissa represents the added noise level, and the ordinate represents the recognition accuracy obtained by the test. It is obvious from Figure 7, Figure 8 and Figure 9 that the recognition accuracy of the blue curve (corresponding to the RCAA-Net method) is higher than other methods. In this work, we can also find the performances of the proposed RCAA-Net dropdown more rapidly when the noise levels increase, which is maybe due to the adverse interaction of various modules when combining them. In the future work, we will explore a more effective combination manner among different modules to further improve the performance for crop disease recognition in actual scenarios. Furthermore, when the noise is added, the recognition accuracy will decrease because the addition of noise will affect the network model extracts and effective features of the image lesions, which in turn affects the accurate recognition of the model. In the same way, the recognition accuracy will decrease as the noise level increases. This is because as the level increases, the number of effective pixels in the lesion area that can be extracted will gradually decrease, which makes it difficult for the model to obtain an accurate prediction category.

It can be seen from Figure 7 that when Gaussian noise with a level of 0.005 is added, the recognition accuracy of all methods gets different degrees of attenuation. Under the conditions of different levels of Gaussian noise, in addition to the RCAA-Net method, the self-attention method has the highest recognition accuracy. When other levels of noise are added, it can also be seen that the RCAA-Net method has the highest recognition accuracy. It can be seen that the anti-interference ability of the RCAA-Net method against Gaussian noise is stronger than other comparison methods. Similar conclusions can be drawn from the test results of adding different noises. Therefore, when adding different levels of noise to the testing set of the 2018ai_challenger crop disease recognition dataset, the proposed RCAA-Net in this paper achieves high accuracy and strong robustness.

## 5. Conclusions

In order to improve the accuracy and robustness of crop disease recognition, this paper introduces a residual self-calibration and self-attention aggregation network (RCAA-Net) for crop disease recognition in actual scenarios. On the one hand, we develop a self-attention aggregation module to automatically focus on the discriminative regions by capturing multi-scale information in different semantic spaces, which can effectively make the proposed RCAA-Net more accurate. On the other hand, we develop a feedback self-calibration module for further suppressing the background noise in the original deep features by fine filtering and adjusting the network features; thereby, effectively improving the effectiveness of the proposed RCAA-Net. Subsequently, in order to verify the proposed RCAA-Net method, this paper carried out corresponding experiments on the 2018ai_challenger crop disease recognition dataset. After a large number of experimental verifications, the proposed RCAA-Net method had higher accuracy and robustness on the same testing dataset. In the next step, we will consider two aspects to further improve the method for crop disease recognition in actual scenarios. Firstly, we plan to add a saliency detection module to the network model to better locate the significant lesion area in the data, further optimize the model structure, and improve the accuracy and robustness of network recognition. Secondly, we will explore a more effective combination manner among different modules to further improve the performance for crop disease recognition in actual scenarios.

## Figures and Tables

**Table 1:** The detailed parameters of the proposed RCAA-Net.

Layers	Output Size
Convolution layer	256×256×16
Residual block1	128×128×32
Residual block2	64×64×64
Residual block3	32×32×128
Feedback self-calibration module 1	128×128×128
Feedback self-calibration module 2	64×64×128
Feedback self-calibration module 3	32×32×128
Self-attention aggregation module	1×128
FC layer	1×256
Softmax layer	1×61

**Table 2:** The comparison results with the state-of-the-art methods.

Methods	Accuracy	Image Size
RCAA-Net	0.892	224×224
RCAA-Net (adaptive)	0.895	256×256
LeafSnap SVM (RBF)	0.407	256×256
LeafSnap NN	0.569	256×256
HCF SVM (RBF)	0.676	256×256
HCF-Scale Robust SVM (RBF)	0.625	256×256
Combined linear SVM	0.871	256×256
SIFT linear SVM	0.548	256×256

**Table 3:** The experimental ablation results.

Methods	Accuracy
RCAA-Net	0.892
Baseline	0.617
RES	0.705
Self-calibration	0.684
Self-attention	0.751

## Data Availability

The datasets generated for this study are available online at https://challenger.ai/competition/pdr2018 (accessed on 5 March 2021).

## References

[1] Wu H., Kang Z., Li X., Li Y., Li Y., Wang S., Liu D. (2020). Identification of Wheat Leaf Rust Resistance Genes in Chinese Wheat Cultivars and the Improved Germplasms. Plant Dis..

[2] Boulent J., Foucher S., Théau J., St-Charles P.L. (2019). Convolutional neural networks for the automatic identification of plant diseases. Front. Plant Sci..

[3] Wang X., Liu J., Zhu X. (2021). Early real-time detection algorithm of tomato diseases and pests in the natural environment. Plant Methods.

[4] Hughes D., Salathé M. (2015). An open access repository of images on plant health to enable the development of mobile disease diagnostics. arXiv.

[5] Guettari N., Capelle-Laizé A.S., Carré P. (2016). Blind image steganalysis based on evidential k-nearest neighbors. Proceedings of the 2016 IEEE International Conference on Image Processing (ICIP).

[6] Deepa S., Umarani R. (2017). Steganalysis on Images using SVM with Selected Hybrid Features of Gini Index Feature Selection Algorithm. Int. J. Adv. Res. Comput. Sci..

[7] Ramezani M., Ghaemmaghami S. (2010). Towards genetic feature selection in image steganalysis. Proceedings of the 2010 7th IEEE Consumer Communications and Networking Conference.

[8] Sheikhan M., Pezhmanpour M., Moin M. (2012). Improved contourlet-based steganalysis using binary ppaper swarm optimization and radial basis neural networks. Neural Comput. Appl..

[9] Kodovsky J., Fridrich J., Holub V. (2011). Ensemble classifiers for steganalysis of digital media. IEEE Trans. Inf. Forensics Secur..

[10] Guo Y., Hastie T., Tibshirani R. (2007). Regularized linear discriminant analysis and its application in microarrays. Biostatistics.

[11] Zhang S., Wang Z. (2016). Cucumber disease recognition based on global-local singular value decomposition. Neurocomputing.

[12] Zhang S., Wu X., You Z., Zhang L. (2017). Leaf image based cucumber disease recognition using sparse representation classification. Comput. Electron. Agric..

[13] Ning H., Zhao B., Yuan Y. (2021). Semantics-Consistent Representation Learning for Remote Sensing Image-Voice Retrieval. IEEE Trans. Geosci. Remote. Sens..

[14] Almabadi E.S., Bauman A., Akhter R., Gugusheff J., Van Buskirk J., Sankey M., Eberhard J. (2021). The Effect of a Personalized Oral Health Education Program on Periodontal Health in an At-Risk Population: A Randomized Controlled Trial. Int. J. Environ. Res. Public Health.

[15] Alsoghair M., Almazyad M., Alburaykan T., Alsultan A., Alnughaymishi A., Almazyad S., Alsuhaibani M. (2021). Medical Students and COVID-19: Knowledge, Preventive Behaviors, and Risk Perception. Int. J. Environ. Res. Public Health.

[16] Duan C., Xiao N. (2021). Parallax-based second-order mixed attention for stereo image super-resolution. IET Comput. Vis..

[17] Xie X., Yang T., Zhang Y., Liang B., Liu L. (2021). Accurate localization of moving objects in dynamic environment for small unmanned aerial vehicle platform using global averaging. IET Comput. Vis..

[18] Hu J., Kong H., Fan L., Zhou J. (2021). Enhancing feature fusion with spatial aggregation and channel fusion for semantic segmentation. IET Comput. Vis..

[19] Kong J., Shen H., Huang K. (2021). DualPathGAN: Facial reenacted emotion synthesis. IET Comput. Vis..

[20] Sohrabi Nasrabadi M., Safabakhsh R. (2021). 3D object recognition with a linear time-varying system of overlay layers. IET Comput. Vis..

[21] Mohanty S.P., Hughes D.P., Salathé M. (2016). Using deep learning for image-based plant disease detection. Front. Plant Sci..

[22] Ma J., Du K., Zheng F., Zhang L., Gong Z., Sun Z. (2018). A recognition method for cucumber diseases using leaf symptom images based on deep convolutional neural network. Comput. Electron. Agric..

[23] Kawasaki Y., Uga H., Kagiwada S., Iyatomi H. (2015). Basic study of automated diagnosis of viral plant diseases using convolutional neural networks. Proceedings of the International Symposium on Visual Computing.

[24] Howard A.G., Zhu M., Chen B., Kalenichenko D., Wang W., Weyand T., Adam H. (2017). Mobilenets: Efficient convolutional neural networks for mobile vision applications. arXiv.

[25] Lin M., Chen Q., Yan S. (2013). Network in network. arXiv.

[26] Ioffe S., Szegedy C. (2015). Batch normalization: Accelerating deep network training by reducing internal covariate shift. PMLR.

[27] He K., Zhang X., Ren S., Sun J. Deep residual learning for image recognition. Proceedings of the IEEE Conference on Computer Vision and Pattern Recognition.

[28] Ding H., Jiang X., Shuai B., Liu A.Q., Wang G. Semantic correlation promoted shape-variant context for segmentation. Proceedings of the IEEE Conference on Computer Vision and Pattern Recognition.

[29] Vaswani A., Shazeer N., Parmar N., Uszkoreit J., Jones L., Gomez A.N., Polosukhin I. (2017). Attention is all you need. arXiv.

[30] Kumar N., Belhumeur P., Biswas A., Jacobs D., Kress W., Lopez I., Soares J. (2012). Leafsnap: A computer vision system for automatic plant species identification. European Conference on Computer Vision.

[31] Hall D., McCool C., Dayoub F., Sunderhauf N., Upcroft B. (2015). Evaluation of features for leaf classification in challenging conditions. Proceedings of the 2015 IEEE Winter Conference on Applications of Computer Vision.

[32] Yang J., Yu K., Gong Y., Huang T. (2009). Linear spatial pyramid matching using sparse coding for image classification. Proceedings of the 2009 IEEE Conference on Computer Vision and Pattern Recognition.

[33] Chang C.C., Lin C.J. (2011). LIBSVM: A library for support vector machines. ACM Trans. Intell. Syst. Technol..

[34] Krizhevsky A., Sutskever I., Hinton G.E. (2012). Imagenet classification with deep convolutional neural networks. Adv. Neural Inf. Process. Syst..

